# Leveraging Large Language Models for Automated Extraction of Abdominal Aortic Aneurysm Features from Radiology Reports

**DOI:** 10.3390/diagnostics16071083

**Published:** 2026-04-03

**Authors:** Praneel Mukherjee, Ryan C. Lee, Roham Hadidchi, Sonya Henry, Michael Coard, Matthew Davis, Yossef Rubinov, Ha Nguyen-Luong, Leah Katz, Tim Q. Duong

**Affiliations:** 1 Department of Radiology, Montefiore Medical Center and Albert Einstein College of Medicine, Bronx, NY 10461, USA; praneel.mukherjee@einsteinmed.edu (P.M.); roham.hadidchi@einsteinmed.edu (R.H.); yossef.rubinov@einsteinmed.edu (Y.R.);; 2 Renaissance School of Medicine, Stony Brook University, Stony Brook, NY 11794, USA

**Keywords:** abdominal aortic aneurysm, large language model, information extraction, radiology report, computed tomography

## Abstract

**Background/Objectives**. Abdominal computed tomography (CT) radiology reports contain critical information for abdominal aortic aneurysm (AAA) management, including aneurysm presence, size, rupture status, and prior repair. However, this information is often embedded within lengthy, heterogeneous reports, making manual extraction inefficient. We evaluated the performance of multiple large language models (LLMs) for automated extraction of AAA-related findings from radiology reports. **Methods**. We retrospectively analyzed 500 abdominal CT reports mentioning AAA from an urban academic health system (2020–2024). Ground truth labels were established by manual review. Four open-source LLMs (Qwen2.5-7B-Instruct, Llama3-Med42-8B, GPT-OSS-20B, and MedGemma-27B-text-it) were evaluated for extraction of aneurysm presence, size, morphology, rupture status, impending rupture, and prior aortic repair. Model outputs were compared with ground truth using exact-match accuracy, and inter-model agreement was assessed using Fleiss’ kappa. Reasoning traces were examined to characterize correct and incorrect model behavior. **Results**. Accuracy for identifying AAA presence ranged from 0.90 to 0.95 (κ = 0.851), and prior aortic repair from 0.90 to 0.97 (κ = 0.793). Accuracy for aneurysm size ranged from 0.67 to 0.88 (κ = 0.340), with low κ’s due to class imbalance or dimension misselection. Rupture and impending rupture were identified with accuracies exceeding 0.90 across models, though agreement was lower (κ = 0.485 and 0.589), reflecting low event prevalence. Larger models (GPT-OSS-20B, MedGemma-27B) generally outperformed smaller models. Reasoning analysis revealed strengths in measurement prioritization but recurrent errors, including dimension misselection, over-inference of prior repair, and conservative classification of rupture-related findings. **Conclusions**. LLMs can accurately extract clinically relevant AAA information from radiology reports with interpretable reasoning, with larger and medically trained models outperforming smaller or general-purpose models. Performance varies by task and model, underscoring the need for careful validation and human-in-the-loop deployment in clinical settings.

## 1. Introduction

Abdominal aortic aneurysm (AAA) is a life-threatening vascular disorder primarily diagnosed and monitored via imaging. Evaluation for AAA is one of the most common indications for abdominal computed tomography (CT) and CT angiography (CTA) [1,2,3,4]. Management is guided by and sensitive to imaging-derived information, particularly aneurysm diameter and growth rate. Current guidelines recommend elective repair when the diameter exceeds 5.5 cm in men or 5.0 cm in women, or when growth exceeds 10 mm per year [2,3,5]; the appropriateness of these intervention thresholds remains a subject of investigation as evidence suggests significant sex-related differences in AAA presentation and surgical outcomes [6,7,8]. CTA abdominal imaging in conjunction with Doppler ultrasound also plays a pivotal role in post-repair surveillance following surgical repair to identify complications such as endoleaks, graft migration, or delayed aneurysm enlargement [6,7].

In routine practice, these imaging derived measurements and features are communicated through the radiology report. For management of most AAA cases, the information required is often limited to a small number of specific findings, such as aneurysm presence, size, growth, or signs concerning for rupture. However, abdominal CTA reports are typically comprehensive and variable in structure, assessing multiple organ systems and findings within a single study. Report organization and phrasing can differ across institutions and even among radiologists within the same practice. As a result, clinically relevant AAA information may be distributed across multiple sections of the report or described using inconsistent language. This variability makes it difficult to reliably identify key findings and complicates both clinical decision making and large-scale research efforts. In most settings, extraction of this information from electronic health records still relies on manual chart review, which is time intensive and poorly scalable.

To address this problem, prior studies have applied natural language processing (NLP) techniques to automate feature extraction from radiology reports obtained for AAA evaluation [9,10,11,12]. While these approaches have shown feasibility, reported performance has been limited. Conventional clinical natural language processing methods, including rule-based systems, conditional random fields (CRF), BiLSTM models, and domain-specific transformers such as ClinicalBERT, are typically designed for narrowly defined tasks and often require extensive feature engineering or task-specific training datasets. In contrast, large language models (LLMs) are large transformer-based models pretrained on massive text corpora using self-supervised learning, allowing them to perform a wide range of language tasks through prompting or minimal fine-tuning. This architecture enables improved contextual understanding and greater flexibility across diverse clinical text analysis tasks compared with traditional NLP pipelines. Traditional NLP models struggle with contextual interpretation and show reduced accuracy when applied across institutions or report styles, particularly in domains with limited labeled data such as vascular imaging.

More recently, large language models such as BERT and GPT have been proposed as an alternative approach. These models are pretrained on large text corpora and can capture contextual relationships within clinical narratives with fewer task specific annotations. However, a significant distinction exists between earlier transformer models like ClinicalBERT, which are primarily encoder-only architectures designed for discriminative tasks and require extensive domain-specific fine-tuning [13], and the modern generative LLMs evaluated in this study. Unlike ClinicalBERT, these larger generative models utilize billions of parameters to enable zero-shot reasoning, allowing for complex information extraction using natural language prompts without the need for additional labeled data. In the setting of AAA imaging, Llama 3.3 70B has been used to extract aneurysm presence and diameter from CTA radiology reports with high accuracy using structured prompting strategies [11]. Other work has shown that unstructured electronic health record text can be used to identify the presence of AAA or repair [14]. An earlier study demonstrated that BERT based models could detect incidental AAAs described in radiology reports [15]. Beyond identification of basic elements such as aneurysm presence or size, LLMs may also be capable of supporting more complex tasks, including construction of AAA cohorts from large health systems [9,10], and interpretation of imaging features that require domain specific knowledge, such as signs concerning for impending rupture or factors relevant to clinical decision making. These more advanced specialized applications, however, have not been systematically evaluated and compared across multiple LLMs in the context of CTA based AAA management [16]. Furthermore, the high level of variability between LLM models, from model size to training data, all may influence the performance of domain specific tasks such as complex AAA information extraction. It is unclear if models with different characteristics will have significantly different performances.

The goal of this study was to evaluate the ability of multiple open-source large language models to automatically extract clinically relevant information about AAA from abdominal CT radiology reports. Using manually reviewed reports as ground truth, we assessed model performance in identifying key features, including aneurysm presence, size, morphology, rupture status, impending rupture, and prior aortic repair. In addition, we compared the performance of several open-source models and analyzed their reasoning traces to better understand model behavior and common errors in radiology report interpretation. We also examined the clinical implications of model reasoning patterns—particularly conservative bias in rupture-related findings—and explored how model scale and domain-specific training influence reasoning capability, with the goal of informing safe integration of LLM-assisted tools into clinical workflows while maintaining human oversight in high-stakes vascular decision-making.

## 2. Materials and Methods

### 2.1. Data Sources

We searched PACS via Montage/Mpower (2020 to 2024) for thoracic and abdominal computed tomography (CT) and CT angiography reports with mentions of AAA. These data were then filtered to include only the most recent 500 reports for analysis. This dataset was enriched with AAA positive cases (not representative of natural incidence) and there was a temporal bias (most recent).

### 2.2. Ground Truth

Review of radiology reports was done by two medical students as this chart review did not require specialized expertise. In case of disagreement, adjudication was done by a board-certified attending. Inter-rater reliability was assessed using Cohen’s or weighted κ. The explicit instructions used by evaluators for determining ground truth are detailed in Table 1. Ground truth labels were assigned based on the explicit language used within the radiology reports. For inferential categories, such as AAA stability or presence of impending rupture, labels were derived from the radiology report’s presence of criteria defined in Table 1 or any explicit concern noted by the radiologist in the report. Only the textual reports written by radiologists were utilized.

### 2.3. LLM Set Up

On the high-performance computing cluster of the Albert Einstein College of Medicine, we initialized Conda, loaded Compute Unified Device Architecture (CUDA) modules, and created a new environment. Within this environment, we installed Python 3.10 and essential Hugging Face packages (v1.0 (0.30+)) for efficient computation. We also installed PyTorch (ver 2.11) with CUDA 12.1 support to enable graphics processing unit (GPU) acceleration, as well as Jupyter Notebook (ver 6.0) for interactive development. After allocating an NVIDIA A100 GPU on the compute node, we launched a Jupyter Notebook session for interactive model inference. We authenticated our Hugging Face account using a personal access token to enable secure access to Qwen2.5-7B-Instruct, Llama3-Med42-8B, GPT-OSS-20B, and MedGemma-27B-text-it. The model and tokenizer were then loaded in batch using the Hugging Face Transformers library, configured for efficient inference with 4-bit quantization and automatic device assignment to the available GPU.

We evaluated four open-source large language models selected to represent diverse architectures and specializations in medical domain tasks. MedGemma-27B and Llama3-Med42-8B were chosen as medical-specific models fine-tuned on clinical corpora, enabling assessment of domain-specialized performance [17,18]. MedGemma-27B represents a larger-scale medical model (27 billion parameters), while Med42-8B offers a more computationally efficient alternative at 8 billion parameters. To benchmark against general-purpose models, we included Qwen2.5-7B-Instruct and GPT-OSS-20B, which are general instruction-tuned models that lack medical pretraining but demonstrate strong reasoning capabilities across domains [19,20]. This selection allows us to examine whether medical domain specialization confers advantages in radiology report comprehension compared to general instruction-following capabilities, while also evaluating the impact of model scale, namely 7–8B against 20–27B, on extraction accuracy across both medical and general model families. To minimize the variance between multiple runs on the same report, each model was set with do_sample = False to force the model into greedy decoding.

### 2.4. LLM Prompting

Each LLM was prompted to extract information from raw radiology reports using the prompting workflow described below. Missing or indeterminate values were labeled as “N/A.” All four models were initially used for information extraction using System Prompt (A) in combination with the input, and reasoning was obtained afterward using System Prompt (B) with the input.

System Prompt (A): You are a radiologist who is an expert at detail extraction within a AAA report. Answer the user’s questions in the order listed. Provide the answer in exactly the user’s format without any additional information. Do not provide any reasoning or explanations.

System Prompt (B): You are a radiologist who is an expert at detail extraction within a AAA report. For the user’s question, first provide brief reasoning (1–2 sentences explaining what you found in the report), then provide your final answer. Format your response as:

Reasoning: [your brief reasoning]

Answer: [your answer in the exact format requested]

Input: Raw radiology report text as context and each of the following questions asked individually, on separate prompts:

- Does this patient have an abdominal aortic aneurysm (AAA)? Only choose your answer from: “yes” or “no”.
- What is the current size of the AAA in centimeters? Provide only the transverse measurement if specified, otherwise extract only the largest measurement. Provide only the numeric value in cm (e.g., “4.5 cm”) or “N/A” if not measurable.
- Is there any evidence of AAA rupture? Only choose your answer from: “yes” or “no”.
- Is there any evidence of impending AAA rupture? Only choose your answer from: “yes” or “no”.
- What is the aneurysm morphology? Only choose your answer from: “fusiform”, “saccular”, or “N/A”.
- Has the AAA remained stable or unchanged over time compared to previous imaging? Only choose your answer from “yes” or “no”, or “N/A” if there is no comparison to previous imaging.
- Does the patient have a history of endovascular aneurysm repair (EVAR) or open aortic repair? Only choose your answer from: “yes” or “no”.

Process: LLM-based extraction of question-specific answers and reasoning per report, following a structured prompt template.

Output: individual_results.csv containing per-report Q&A pairs and structured reasoning statements.

### 2.5. Statistical Analysis

Information extracted by all LLMs were evaluated for accuracy on a per-report and per-question basis. Binary classifications were utilized for accuracy in “yes/no” questions whereas exact measurement accuracy was utilized for LLM extraction of the aneurysm size. As “size,” “impending rupture,” and “rupture” ground truths were only extracted in the case of a true AAA, statistical metrics such as sensitivity and specificity were only calculated against AAAs that were correctly identified. Confusion matrices were used to visualize the error of each LLM on questions with binary answers. Fleiss’ kappa was calculated to evaluate for inter-LLM variability of LLM performance on individual reports. A set of pairwise statistical comparisons were then made using McNemar’s test to evaluate for difference in performance between large vs small sized models and domain-specific vs general-purpose models. The specific comparisons made were those between MedGemma-27B and GPT-OSS-20B, Qwen2.5-7B-Instruct and GPT-OSS-20B, MedGemma-27B and Llama3-Med42-8B, and Llama3-Med42-8B and Qwen2.5-7B-Instruct. We then analyzed the reasoning of LLMs to report on their ability to interpret complex medical language and report examples of positive and negative performance.

## 3. Results

### 3.1. Ground Truth Characteristics

The ground truth characteristics determined by chart review is shown in Table 2. Out of the 500 reports, 204 (40.8%) contained at least one AAA. Of those with AAA, the average size was 4.9 cm. There were seven (3.4%) active rupture cases and 22 (10.8%) impending rupture cases amongst all reports with AAA. There were 117 (23.4%) reports with prior aortic repair across all reviewed reports. Aneurysm morphology was sparsely reported. There were 52 (25.5%) reports explicitly noting a fusiform morphology and 14 (6.9%) noting a saccular/eccentric aneurysm, with 138 (67.6%) containing no mention of morphology.

Inter-rater agreement is shown in Table 3. There was substantial agreement among annotations, with Cohen’s or quadratic weighted κ ranging from 0.895 to 0.987.

### 3.2. LLM Performance

LLMs were used to extract the presence of AAA, AAA size, rupture status, impending rupture status, prior aortic repair status, and AAA morphology for each of the 500 reports, and to compare with the ground truths. Table 4 summarizes the extracted cohort characteristics determined by each LLM for the 500 radiology reports. Models identified AAA presence in 38.8 to 41.4% of reports (mean 40.1 ± 1.2%). Mean AAA size estimates ranged from 5.0 to 6.6 cm across models. Ruptured AAA prevalence ranged from 1.0% to 6.8% (3.1 ± 2.6%), and impending rupture ranged from 2.1% to 11.2% (mean 6.6 ± 3.9%). Prior repair was identified in 13.4% to 24.6% of all reports (mean 21.2 ± 5.3%). Fusiform morphology was detected in 14.4 to 42.5% of AAA cases, (mean 30.4 ± 11.8%), whereas saccular morphology was detected in 8.7 to 25.9% of cases (mean 15.1 ± 7.6%). Morphology was not detected in 37.6 to 74.7% of cases (mean 54.5 ± 17.3%).

**Figure 1:**
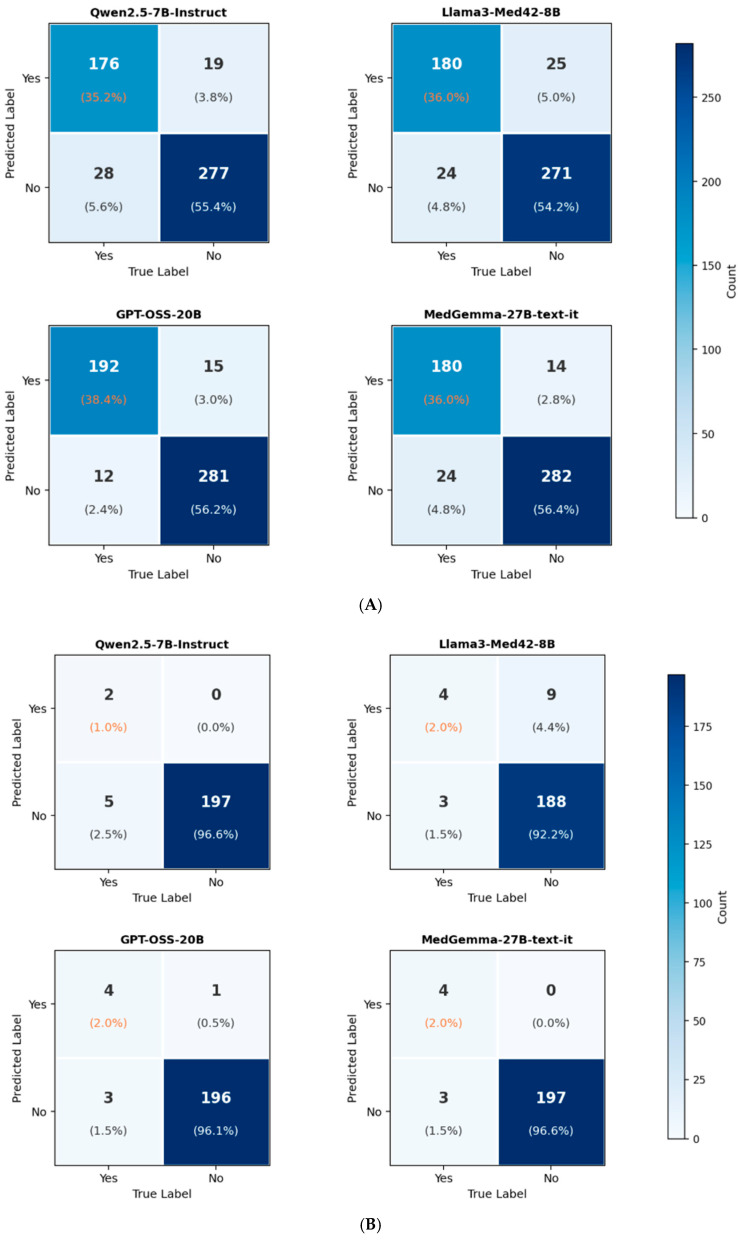

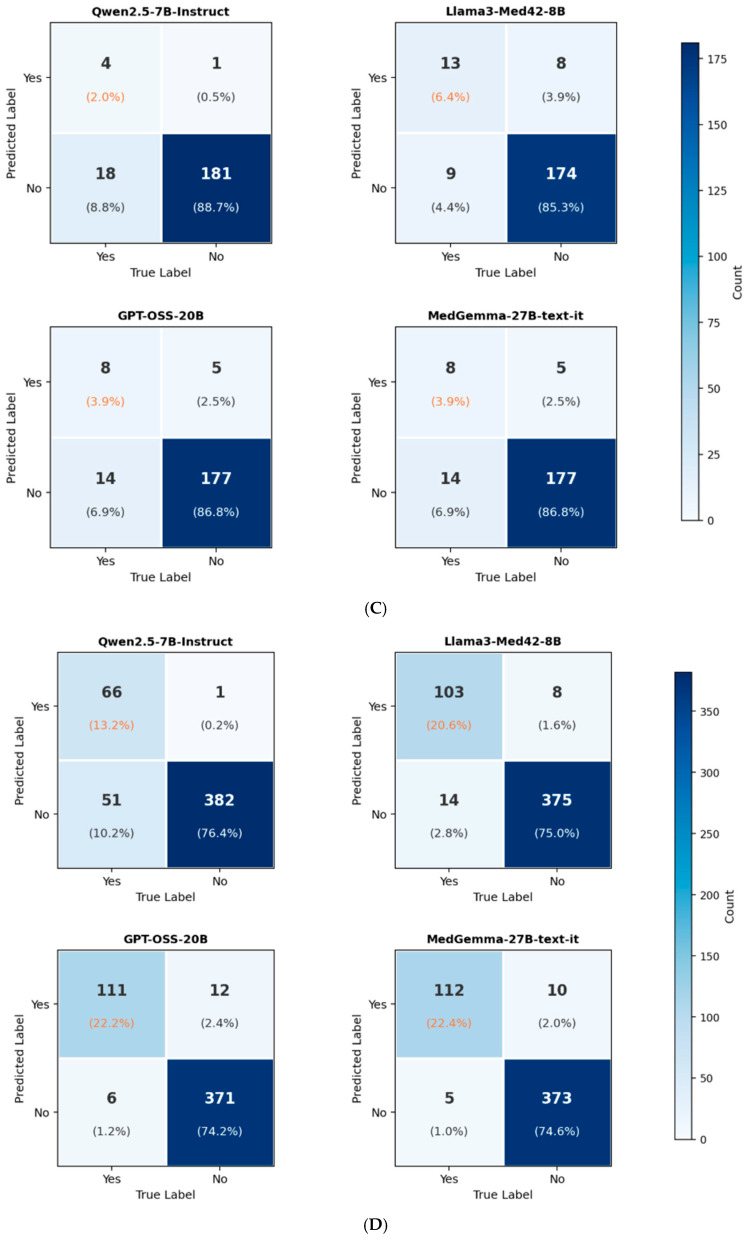
Confusion matrix for LLM identification of (**A**) the presence of AAA, (**B**) rupture, (**C**) impending rupture, and (**D**) prior repair using Qwen2.5-7B-Instruct (upper left), Llama3-Med42-8B (upper right), GPT-OSS-20B (lower left), and MedGemma-27B-text-it (lower right). AAA presence and prior repair were computed against all 500 reports, while rupture and impending rupture were computed against the subset of 204 reports that have AAA from the ground truths.

Figure 1 shows the confusion matrix for presence of AAA, rupture, impending rupture, and prior repair for the 4 evaluated LLMs.

Table 5 summarizes the accuracies for all four LLMs’ identification of the presence of AAA, AAA size, rupture status, impending rupture status, and prior aortic repair status. The accuracies for all variables were high. The average accuracy across categories ranged from 0.91 to 0.97, except that of size, which was 0.78. GPT-OSS-20B and MedGemma-27B-text-it performed similarly, and both performed qualitatively better than Qwen2.5-7B-Instruct and Llama3-Med42-8B, with these two models also performing similarly. The LLM with the highest average performance across all five categories, GPT-OSS-20B, identified patients with AAA with 94.6% accuracy, size of AAA with 88.2% accuracy, prior repair with 96.4% accuracy, rupture with 98% accuracy, and impending rupture with 90.7% accuracy.

The variables with the highest agreement amongst the models were AAA presence (Fleiss’ kappa = 0.851) and prior repair (Fleiss’ kappa = 0.793). The variables with the lowest agreement were rupture (Fleiss’ kappa = 0.485) and impending rupture (Fleiss’ kappa = 0.589). The low agreement was because of low incidence of those variables.

McNemar’s test identified significant performance differences between model pairs for specific tasks. Larger sized models (GPT-OSS-20B and MedGemma-27B-text-it) generally performed better compared to smaller sized models (Qwen2.5-7B-Instruct and Llama3-Med42-8B). Amongst the smaller sized-models, the domain specific model (Llama3-Med42-8B) generally performed better compared to its general purpose, smaller sized counterpart (Qwen2.5-7B-Instruct).

Table 6 presents a detailed evaluation of model performance across four primary clinical tasks: AAA presence, rupture, impending rupture, and prior repair. Metrics were derived from the raw counts in Figure 1 to assess clinical utility beyond simple accuracy. Note the high specificity across tasks contrasted by lower sensitivity in rupture-related categories, quantitatively illustrating the conservative bias identified in model reasoning traces.

### 3.3. Reasoning

We investigated the reasoning capability across all four LLMs by extracting the underlying logic used to generate their responses, with representative excerpts from GPT-OSS-20B provided. In instances of correct extraction (Table 7A), the model accurately identified a 7.5 cm bilobed infrarenal aneurysm, correctly prioritizing the largest transverse diameter from the listed dimensions. Its reasoning demonstrated a nuanced understanding of acute pathology, as it correctly concluded that the aneurysm was not ruptured or an impending rupture due to the absence of periaortic inflammation, extravasation, or free fluid, despite the high-risk size. Furthermore, the model correctly reasoned that the absence of specific surgical documentation in the report indicated no prior history of EVAR or open aortic repair. Common errors in model-derived size extraction include pulling an older measurement that is not being asked for, selecting incorrect anatomical axes for measurement, and mixing up multiple measurements in the report. Downstream clinical users should verify LLM-extracted measurements against the report section to ensure the most current and relevant measurement is prioritized.

We also analyzed instances where the models provided incorrect reasoning, leading to discrepancies with the ground truth (Table 7B). The models exhibited failures in dimension prioritization, such as extracting a 7.6 cm measurement from the “Vessels” section when a larger 8.5 cm dimension was listed in the “Impression.” There was also a notable tendency to over-infer surgical history, such as incorrectly concluding that the presence of iliac stents served as definitive evidence of a prior EVAR. Most notably, the reasoning regarding rupture was often overly conservative: while the models identified findings such as mural thrombus, endoleaks, or significant diameter increases, they incorrectly reasoned that these did not constitute acute pathology or impending rupture in the absence of “classic” secondary signs like active contrast extravasation.

## 4. Discussion

This study is among the first to evaluate LLMs for extracting complex abdominal aortic aneurysm findings from radiology reports. LLMs achieved high accuracy for aneurysm presence (up to 94.6%) and size (up to 88.2%), with larger and medically trained models outperforming smaller or general-purpose models. Lower performance for active or impending rupture likely reflected the rarity of true positive cases. Although LLMs demonstrated contextual reasoning, errors occurred in dimension prioritization, prior repair inference, and conservative interpretation of rupture-related findings. Overall, these results demonstrate the feasibility of LLM usage in retrospective controlled settings within vascular imaging.

The use of LLMs in this study demonstrate a high degree of accuracy overall and offer comparable performance to previous usages of NLP in AAA detection [21]. Conventional NLP techniques relied heavily on rule-based systems or task-specific machine learning models that required substantial manual annotation and rule engineering, often involving 400 to 4870 manually annotated radiology reports to achieve high performance [10,22]. In comparison, the LLM-based approach in the present study demonstrated similarly high accuracy while requiring less manual feature engineering, highlighting the potential of LLMs to streamline clinical text analysis and improve scalability for radiology report interpretation.

Analysis of inter-model agreement (Fleiss’ kappa) further validated these results, showing strong consensus for AAA presence (κ = 0.851) and prior repair (κ = 0.793), supporting their suitability for automated workflows. Notably, however, the moderate kappa for rupture status (κ = 0.485) reflects the impact of class imbalance. With only seven ruptured cases among the 204 reports with AAA (3.4% prevalence in the AAA cohort), the four models had 0% agreement correctly on three reports, 75% agreement correctly on two reports, and 100% agreement correctly on two reports. This shows that the models faced difficulty in identifying AAA rupture, as only four of the seven rupture cases experienced an agreement rate of at least 75%, highlighting how the four LLMs have relatively low performance for rare but clinically critical events like rupture. Furthermore, AAA size measurement showed only fair, relatively lower agreement (κ = 0.340), indicating that precise numerical extraction remains variable and may require human oversight or further prompt engineering.

Our analysis showed that models frequently defaulted to negative predictions for low prevalence but high-risk categories. For example, the detection rate for “impending rupture” (2.1–11.2%) was generally lower than the ground truth prevalence of 10.8%. Analysis of reasoning traces reveals that this stems from a failure to synthesize clinical indicators into a higher-level risk assessment. In one case, a model correctly identified a significant aneurysm expansion (from 5.3 to 8.5 cm) and a possible endoleak but incorrectly concluded there was no impending rupture simply because “classic” signs like active extravasation were absent. This behavior highlights how while LLMs are effective at extracting explicit keywords, they struggle with the nuance required to identify unstable pathology in the absence of definitive terminology. Furthermore, it highlights a consistent trend in how modern LLMs tend to be overly conservative in judgement, resulting in lower recall scores [23]. Unlike false positives, which primarily result in unnecessary follow-up costs, these false negatives pose a direct patient safety risk by potentially delaying urgent intervention. Furthermore, because our dataset was enriched for AAA cases (40.8%) compared to general screening populations (1–5%), this conservative bias suggests that in real-world deployment, LLMs might miss an unacceptable proportion of the rare, life-threatening events they are intended to flag.

A key finding of this study is the influence of model architecture on extraction performance. We observed that model scale played a significant role, with larger models (GPT-OSS-20B and MedGemma-27B) generally outperforming their smaller counterparts (Qwen2.5-7B and Llama3-Med42-8B) across most tasks. However, domain specialization appeared to mitigate some limitations of smaller model size. Among the smaller models, the medically fine-tuned Llama3-Med42-8B generally performed better than the general-purpose Qwen2.5-7B-Instruct. These results are consistent with prior reports that showed that the 70B model of Llama outperformed the 8B model [24] and the specialized GPT-4o outperformed base GPT-4o [25]. This suggests that for resource-constrained environments where deploying massive models is not feasible, smaller models fine-tuned on clinical corpora may offer a viable alternative. Nevertheless, for complex inferential tasks in vascular imaging, larger parameter counts appear necessary to maximize accuracy and reasoning capability. A notable finding was the significant performance deviation of Llama3-Med42-8B in numerical extraction, which estimated a mean AAA size of 6.6 cm compared to the ground truth of 4.9 cm. While domain-specific fine-tuning often enhances clinical reasoning, this specific medical model likely suffered from “hallucinatory inflation” or a tendency to prioritize high-risk, maximum dimensions mentioned in training corpora over the precise transverse measurements requested in our prompt. Unlike general-purpose models like Qwen2.5-7B, which adhered strictly to the numerical values provided in the text, the medically fine-tuned Llama3-Med42-8B model may have failed to distinguish between current findings and historical maximums often cited in vascular reports. This underscores that while medical specialization improves task-specific terminology, it does not inherently guarantee superior accuracy in precise numerical data extraction without extensive instruction-tuning for quantitative tasks.

The quantitative analysis of clinical metrics reveals a significant discrepancy between model precision and recall, particularly for high-stakes findings. For the detection of active rupture, models exhibited near-perfect specificity (0.954–1.000) and high precision (0.308–1.000), resulting in low false-positive rates. However, this was accompanied by a markedly elevated false-negative rate, with sensitivity ranging from 0.286 to 0.571. A similar trend was observed for impending rupture, where the highest achieved sensitivity was only 0.591. In contrast, for high-prevalence categories like AAA presence and prior repair, the models demonstrated a more balanced performance profile, with precision for both categories exceeding 0.90 across both larger models and recall exceeding 0.88. These metrics quantitatively confirm that the conservative bias identified in reasoning traces directly translates to a high rate of missed clinical events in low-prevalence, high-risk scenarios.

Finally, model scale significantly influenced computational efficiency and inference latency. In our evaluation, Qwen2.5-7B-Instruct demonstrated the highest throughput with the shortest inference times, followed sequentially by Llama3-Med42-8B, GPT-OSS-20B, and MedGemma-27B. While the larger 20-27B parameter models achieved superior extraction accuracy, their increased computational requirements and higher latency present challenges for real-time clinical integration. Conversely, the high-speed performance of the 7–8B models suggests they are better suited for high-volume batch processing of historical archives. These findings indicate that the selection of an LLM for radiology workflows must balance the trade-off between the absolute accuracy of larger architectures and the operational scalability of smaller, more efficient models.

### Limitations and Future Directions

This study has several limitations. This study used data from a single institution, which may limit the generalizability of the findings to other clinical settings with different patient populations, imaging protocols, or documentation practices. External validation using multi-institutional datasets will be necessary to assess the robustness and broader applicability of the proposed approach. Future work should therefore focus on validating these methods across diverse healthcare systems and prospective clinical environments.

Second, the limited number of rare but clinically critical cases, such as active or impending rupture, constrained performance evaluation and likely contributed to lower agreement, emphasizing the need for larger, more diverse datasets. Model performance was also sensitive to prompt formulation, highlighting the importance of systematic prompt design [26]. To mitigate the safety risks associated with missed “impending rupture” cases, future workflows could employ specialized “high sensitivity” prompting or multi-agent Chain of Thought (CoT) architectures. In a high-sensitivity framework, the system prompt is modified to explicitly instruct the model to prioritize clinical suspicion over definitive terminology, lowering the threshold for flagging potentially unstable findings. Alternatively, a multi-agent approach, where one agent extracts explicit findings and a second “critic” agent performs a high-level risk synthesis, may better replicate the expert radiologist’s ability to infer impending rupture from subtle signs like thrombus fissuration or rapid diameter expansion. These strategies, combined with uncertainty-aware clinician oversight, represent a robust path toward reducing false negatives in high-stakes vascular management.

While this study demonstrates successful LLM-based extraction for reports directly involving AAA, performance may vary in a general clinical workflow where the prevalence of the condition is significantly lower. Future validation should utilize unselected, consecutive cohorts from multiple institutions to ensure that models maintain high specificity and mitigate the risk of algorithmic bias stemming from site-specific reporting templates. Beyond technical performance, the clinical deployment of these models introduces critical ethical implications regarding accountability and automation bias. If clinicians over-rely on automated summaries, the conservative bias, where models missed life-threatening findings due to a lack of explicit terminology, could lead to delayed interventions and direct patient harm. These tools must be implemented as decision-support systems that maintain a human-in-the-loop to ensure transparency and clinical oversight in high-stakes vascular management. Interpretability and potential bias remain challenges for clinical translation. Although reasoning outputs improve transparency, errors in measurement prioritization, inference of prior repair, and conservative interpretation of rupture-related findings persist, highlighting the need for domain-specific refinement and uncertainty-aware clinician oversight.

Prospective, workflow-based studies are needed to assess the real-world impact of LLM-assisted extraction on efficiency and clinical decision-making. Ongoing refinement using multi-institutional data and expert feedback will be essential, with LLMs best positioned as decision-support tools that augment radiologist interpretation rather than replace it.

## 5. Conclusions

This study shows that LLMs accurately extract abdominal aortic aneurysm features from radiology reports (88.2–98.5%) without extensive task-specific training. However, the observed conservative bias across all models can lead to the under-reporting of rare high-risk findings when explicit language is absent. Larger and medically trained models generally outperform smaller or general-purpose models. These results point to the potential of LLM use for registry building and workflow triage, while underscoring the need for human-in-the-loop oversight for high-stakes clinical decisions.

## Figures and Tables

**Table 1:** Questions and instructions for ground truth determination.

*Question/Information*	*Extraction Instructions*
Is there an abdominal aortic aneurysm in the radiology report?	Y/N; thoracic and iliac aneurysms are explicitly excluded and would result in N.
If aneurysm present, what is the size of the largest aneurysm?	Size in cm; in the largest aneurysm, extract the measurement of the transverse axis. If the transverse axis is not specified, extract the largest measurement.
If aneurysm present, what is the morphology of the aneurysm?	Fusiform/saccular/eccentric/NA
If aneurysm present, has the aneurysm remained stable over time?	Y/N/NA; if the radiologist explicitly states that the size has remained stable or been unstable, then that determines the rating. If no explicit mention, then growth < 0.5 cm over a year is rated as stable. If no comparison given, then N/A.
If aneurysm present, are there signs of rupture?	Y/N; if the radiologist reported explicitly that it appeared the aneurysm had active rupture
If no active rupture, are there signs of impending rupture?	Y/N; if the presence of any of these findings was reported, it was considered a sign of impending rupture: increased aneurysm size on serial imaging (rate of 10 mm or more), aneurysm > 7 cm on transverse axis, reduced thrombus size, thrombus fissuration, discontinuity in calcification, high-attenuation crescent sign.
Were there any prior endovascular or open repairs?	Y/N; if any prior EVAR or open repair was made on either thoracic or abdominal aorta.

**Table 2:** Ground truth characteristics determined by manual chart review for the 500 analyzed reports. * Within a real-world clinical context, most AAA are likely fusiform in nature. However, ground truths were only extracted for explicit mentions of a distinctive morphology to prevent inferences.

Cohort Ground Truths (*N* = 500)	
AAA presence	40.8% (204/500)
Mean AAA size (cm)	4.9
Ruptured	3.4% (7/204)
Impending rupture	10.8% (22/204)
Prior repair	23.4% (117/500)
Morphology (*N* = 204)	
Fusiform	25.5% (52/204)
Saccular/eccentric	6.9% (14/204)
Not reported *	67.6% (138/204)

**Table 3:** Statistics for inter-reader variability for ground truth annotations. Cohen’s κ was utilized for all categories other than the size category where quadratic weighted κ was utilized. A confidence interval of 95% is indicated in the brackets.

Task	κ
AAA presence	0.948 [0.911–0.986]
Mean AAA size	0.987 [0.956–1.000]
Ruptured	0.905 [0.719–1.000]
Impending rupture	0.895 [0.778–1.000]
Morphology	0.987 [0.961–1.000]

**Table 4:** AAA features extracted by LLMs. Note that total prediction counts for conditional variables (rupture/impending rupture) may differ slightly between Table 4 and Figure 1, as Figure 1 restricts analysis exclusively to the 204 ground-truth confirmed AAA cases. * Value in parenthesis represents number of reports from which the model successfully extracted a size measurement.

Criteria	Qwen2.5- 7B-Instruct	Llama3-Med42-8B	GPT-OSS- 20B	MedGemma- 27B-Text-It
AAA present	39.0% (195/500)	41.0% (205/500)	41.4% (207/500)	38.8% (194/500)
Average AAA size *	5.1 cm (192)	6.6 cm (196)	5.0 cm (200)	5.0 cm (191)
Ruptured	1.0% (2/195)	6.8% (14/205)	2.4% (5/207)	2.1% (4/194)
Impending rupture	2.1% (4/195)	11.2% (23/205)	6.3% (13/207)	6.7% (13/194)
Prior repair	13.4% (67/500)	22.2% (111/500)	24.6% (123/500)	24.4% (122/500)
Morphology				
Fusiform	28.2% (55/195)	36.5% (75/205)	42.5% (88/207)	14.5% (28/194)
Saccular/eccentric	8.7% (17/195)	25.9% (53/205)	15.0% (31/207)	10.8% (21/194)
Not reported	63.1% (123/195)	37.6% (77/205)	42.5% (88/207)	74.7% (145/194)

**Table 5:** LLM model accuracy. Accuracy was calculated at the report level using exact-match criteria. AAA presence and prior repair were evaluated against all 500 reports from the ground truths, whereas AAA size, rupture, and impending rupture were evaluated against the subset of 204 reports that have AAA from the ground truths. Superscript symbols indicate pair-wise comparison using McNemar’s test which reached significance (*p* < 0.05) in difference of accuracy. ^#^ indicates a significant difference between Qwen2.5-7B-Instruct and GPT-OSS-20B. ^$^ indicates a significant difference between GPT-OSS-20B and MedGemma-27B-text-it. ^^^ indicates a significant difference between Llama3-Med42-8B and MedGemma-27B-text-it. * indicates a significant difference between Qwen2.5-7B-Instruct and Llama3-Med42-8B. Fleiss’ Kappa indicates agreement across 4 models.

Category	Qwen2.5- 7B-Instruct	Llama3-Med42-8B	GPT-OSS- 20B	MedGemma 27B-Text-It	Fleiss’ Kappa
AAA presence	0.906 ^#^	0.902	0.946 ^$,#^	0.924 ^$^	0.851
AAA size	0.691 ^#^	0.667 ^^^	0.882 ^#^	0.868 ^^^	0.340
Rupture	0.975	0.941	0.98	0.985	0.485
Impending rupture	0.907	0.917	0.907	0.907	0.589
Prior repair	0.896 *^,#^	0.956 *	0.964 ^#^	0.97	0.793

**Table 6:** Comprehensive performance metrics for AAA feature extraction across four large language models. * Rupture and impending rupture metrics were calculated over the subset of 204 AAA cases from the ground truths and not LLM derived AAA cases, as these are conditional fields.

Task & Model	Sensitivity	Specificity	PPV	NPV	F1 Score
**AAA presence**					
Qwen2.5-7B-Instruct	0.863	0.936	0.903	0.908	0.882
Llama3-Med42-8B	0.882	0.916	0.878	0.919	0.880
GPT-OSS-20B	0.941	0.949	0.928	0.959	0.934
MedGemma-27B	0.882	0.953	0.928	0.922	0.905
Rupture *					
Qwen2.5-7B-Instruct	0.286	1	1	0.975	0.444
Llama3-Med42-8B	0.571	0.954	0.308	0.984	0.4
GPT-OSS-20B	0.571	0.995	0.8	0.985	0.667
MedGemma-27B	0.571	1	1	0.985	0.727
Impending rupture *					
Qwen2.5-7B-Instruct	0.182	0.995	0.8	0.910	0.296
Llama3-Med42-8B	0.591	0.956	0.619	0.951	0.605
GPT-OSS-20B	0.364	0.973	0.615	0.927	0.457
MedGemma-27B	0.364	0.973	0.615	0.927	0.457
**Prior repair**					
Qwen2.5-7B-Instruct	0.564	0.997	0.985	0.882	0.717
Llama3-Med42-8B	0.88	0.979	0.928	0.964	0.904
GPT-OSS-20B	0.949	0.969	0.902	0.984	0.925
MedGemma-27B	0.957	0.974	0.918	0.987	0.937

**Table 7:** Examples of (**A**) correct and (**B**) incorrect reasoning and answers for GPT-OSS-20B.

(A) Question	Reasoning	Model Answer	Ground Truth
Does this patient have an abdominal aortic aneurysm (AAA)?	The report identifies a bilobed infrarenal aneurysm measuring 7.5 × 4.6 × 4.2 cm, confirming the presence of an abdominal aortic aneurysm.	Yes	Yes
What is the current size of the AAA in centimeters?	The report lists the aneurysm dimensions as 7.5 × 4.6 × 4.2 cm, with the largest (transverse) measurement being 7.5 cm.	7.5 cm	7.5 cm
Is there any evidence of AAA rupture?	The report notes a bilobed infrarenal aneurysm with slight increase in size and mural thrombus extending to the diaphragmatic hiatus, but there are no findings of free fluid, extravasation, or other signs of rupture.	No	No
Is there any evidence of impending AAA rupture?	The aneurysm measures 7.5 cm in diameter, which is above the threshold for high risk, but there are no acute signs of impending rupture such as rapid expansion, intraluminal thrombus rupture, or periaortic inflammation.	No	No
Does the patient have a history of endovascular aneurysm repair (EVAR) or open aortic repair?	The report contains no mention of prior endovascular aneurysm repair (EVAR) or open aortic repair procedures.	No	No
**(B) Question**	**Reasoning**	**Model Answer**	**Ground Truth**
Does this patient have an abdominal aortic aneurysm (AAA)?	The report notes a large aneurysm (up to 3 cm) within the abdominal aortic endograft, indicating an aneurysmal dilation in the abdominal aorta.	Yes	No
What is the current size of the AAA in centimeters?	The report states the aneurysm measures 7.6 × 7 cm; the largest dimension is 7.6 cm.	7.6 cm	8.5 cm
Is there any evidence of AAA rupture?	The report describes an infrarenal abdominal aortic aneurysm measuring up to 3.2 cm with mural thrombus but shows no evidence of free fluid, extravasation, or other classic signs of rupture.	No	Yes
Is there any evidence of impending AAA rupture?	The aneurysm has enlarged to 8.5 cm and shows possible endoleak, but there is no active extravasation, pericardial effusion, or other classic signs of imminent rupture.	No	Yes
Does the patient have a history of endovascular aneurysm repair (EVAR) or open aortic repair?	The report notes bilateral common iliac stents, which are indicative of prior endovascular aneurysm repair (EVAR).	Yes	No

Note: Example B (incorrect reasoning) includes questions and responses applied to multiple reports, while Example A answers all come from the same report. This was done because the model did not provide all incorrect answers for a single report, and the reasoning is self-contained for each question.

## Data Availability

The data presented in study are available on request from the corresponding author.
